# 

**Published:** 1848-10

**Authors:** 


### CONTENTS

OF THE

### DENTAL REGISTER OF THE WEST.

### OCTOBER, 1848.

Art. 1. Observations on Hæmorrhage, by Dr. B. B. Brown, St. Louis Editor . . . . . . . . Page 1
" 2. Fourth Annual Meeting of the M. V. A. of Dental Surgeons . . . . . . . . 18
" 3. Essay on Pivot Teeth, by Chas. Bonsall, D. D. S. . . 25
" 4. Synopsis of Discussions at Fourth Annual Meeting of the M. V. A. D. S. . . . . . . . . 28
" 5. The Use of the Key, by Dr. J. Taft . . . . . . . . 35
" 6. Letter from Dr. Stratton, St. Louis, Mo. . . . . . . . . 40
" 7. Letter from Dr. Haven, " " . . . . . . . . 43
" 8. Hill's Stopping, by St. Louis Editor . . . . . . . . 45
" 9. Dental Advertisements, by Cin. Editor . . . . . . . . 46
" 10. Modified Tooth Key, by St. Louis Editor . . . . . . . . 47
" 11. New York Dental Recorder, by St. Louis Editor . . . . 48
" 12. White's Toothache Drops . . . . . . . . 49
" 13. General Index for first Volume . . . . . . . . 50
" 14. Third Dentition—Vicar. Mens . . . . . . . . 50
" 15. To Our Correspondents . . . . . . . . 50
" 16. To Subscribers . . . . . . . . 51
" 17. American Journal and Library of Dental Science . . . 51
" 18. Obituary . . . . . . . . 51

